# Contribution of Ruminal Bacteriome to the Individual Variation of Nitrogen Utilization Efficiency of Dairy Cows

**DOI:** 10.3389/fmicb.2022.815225

**Published:** 2022-03-18

**Authors:** Min Li, Huiyue Zhong, Ming Li, Nan Zheng, Jiaqi Wang, Shengguo Zhao

**Affiliations:** State Key Laboratory of Animal Nutrition, Institute of Animal Sciences, Chinese Academy of Agricultural Sciences, Beijing, China

**Keywords:** rumen, nitrogen utilization efficiency, bacteriome, dairy cows, milk

## Abstract

High nitrogen utilization efficiency (NUE) is important for increasing milk protein production and decreasing the feed nitrogen cost and nitrogen emission to the environment. Currently, there is a limited whole picture of the relationship between ruminal bacteriome and the NUE of dairy cows, even though some information has been revealed about the bacteriome and milk or milk protein production of dairy cows. The purpose of this study was to compare the rumen bacterial community in dairy cows with different nitrogen utilization efficiency under the same diet. The natural abundance of ^15^N between the animal proteins and diet (Δ^15^N) was used as a simple, non-invasive, and accurate biomarker for NUE in ruminants to mark the individual variation. Dairy cows with high NUE (HE_HP, *n* = 7), medium NUE (ME_MP, *n* = 7), and low NUE (LE_LP, *n* = 7) were selected from 284 Holstein dairy cows with the same diet. Measurement of the rumen fermentation indices showed that the proportion of propionate was higher in HE_HP cows and ME_MP cows than in LE_LP cows (*P* < 0.05). The diversity of rumen bacterial community was higher in LE_LP cows than in ME_MP cows and HE_HP cows by 16S rRNA sequencing analysis (*P* < 0.05). Moreover, at the genus level, the relative abundances of *Succinivibrionaceae*_UCG_001, uncultured_*Selenomonadaceae*, and *Acidaminococcus* were higher in HE_HP cows than in LE_LP cows (*P* < 0.05). Interestingly, we found that these bacteria were positively correlated with milk protein yield and negatively correlated with Δ^15^N (*P* < 0.05). However, *Clostridia*_UCG_014, *Saccharofermentans*, *Bacilli*_RF39, and *Desulfovibrio* were lower in HE_HP cows and ME_MP cows than in LE_LP cows (*P* < 0.05), which were negatively correlated with milk protein yield and positively correlated with Δ^15^N (*P* < 0.05). In conclusion, the study showed that the diversity and relative abundances of rumen bacteria differed among different NUE cows, indicating that rumen bacteriome contributes to nitrogen metabolism in dairy cows.

## Introduction

With the development of animal husbandry, most of the nitrogen intake by dairy cows is excreted by feces and urine. Nitrogen excretion leads to emission of greenhouse gases by livestock, followed by nitrogen loss in soil (N_2_O), volatilization of NH_3_, and leaching of NO_3_^–^, to environment pollution, aquatic and terrestrial eutrophication, and acidification (Angelidis et al., 2019). Oenema and Tamminga (2005) showed that the global amount of nitrogen annually excreted by livestock ranged from 8 × 10^7^ T to 1.3 × 10^8^ T, and the production of cattle contributed 60% of the total emissions. Therefore, it is necessary to improve the nitrogen utilization efficiency (NUE) of dairy cows by dietary regulation. NUE can be affected by heredity, nutrition, and management (Wang et al., 2007; Broderick et al., 2008; Cantalapiedra-Hijar et al., 2014)(Broderick et al., 2008; Wang et al., 2007; Cantalapiedra-Hijar et al., 2014). It was reported that the NUE of dairy cows was still at a low level (only ∼25%) (Huhtanen and Hristov, 2009), and more nitrogen was emitted to the environment (Calsamiglia et al., 2010).

Stable isotope fractionation, a result of the change in the ratio of heavy to light isotope (^15^N/^14^N), can be used to characterize the nutrition status of animals (Lavery and Ferris, 2021). δ^15^N is the ratio of ^15^N/^14^N in the animal minus ^15^N/^14^N ratio in the standard. N partitioning is different in plasma, feces, and milk compared to diet. It was reported that N isotope fractionation can predict NUE (Cheng et al., 2013). Plasma nitrogen isotopic fractionation (Δ^15^N = δ^15^N_*plasma*_ - δ^15^N_*diet*_) can be especially used as a predictor to effectively assess NUE (Cheng et al., 2016). Cantalapiedra-Hijar et al. (2018) suggested that Δ^15^N could be used to predict NUE variation across diets and individuals. Δ^15^N was demonstrated to be negatively correlated with NUE and the synthesis of microbe protein under different conditions (Cheng et al., 2016).

Rumen microbiota can convert fiber into digestible substances such as volatile fatty acid (VFAs) and microbial proteins. Ruminal VFAs produced by rumen microbial fermentation can provide 70% of the energy requirements in dairy cows (Matthews et al., 2019). Moreover, the bacteria in the rumen accounts for more than 95% of the microbial community, which is the main contributor to the performance of dairy cows (Castillo-Gonzalez et al., 2014). Li et al. (2019) found that the microbial composition and function between high residual feed intake steers and low residual feed intake steers were different, suggesting that microbiome interactions are the major contributor to the variations of feed efficiency. Xue et al. (2018) revealed that pan and core bacteriome were associated with milking traits in lactating dairy cows which were fed the same diet under the same environment, and they also found that the abundance of *Sharpea* was higher in high-milk-protein-yield (MPY) cows than in low-MPY cows. *Succinivibrio* and *Clostridium* were enriched in low-MPY cows compared to high-MPY cows. Although specific bacteria were related to milk composition (Jami et al., 2014), there are few studies on uncovering the function of rumen bacteria to NUE. The interaction of rumen bacteria and NUE is not clear.

In this study, Δ^15^N and MPY, as NUE indicators, were used to discuss NUE’s relationship with the rumen bacteriome. The objective of the study was to reveal the potential effects of rumen bacteriome on NUE in dairy cows.

## Materials and Methods

### Animals and Experimental Design

**FIGURE 1:**
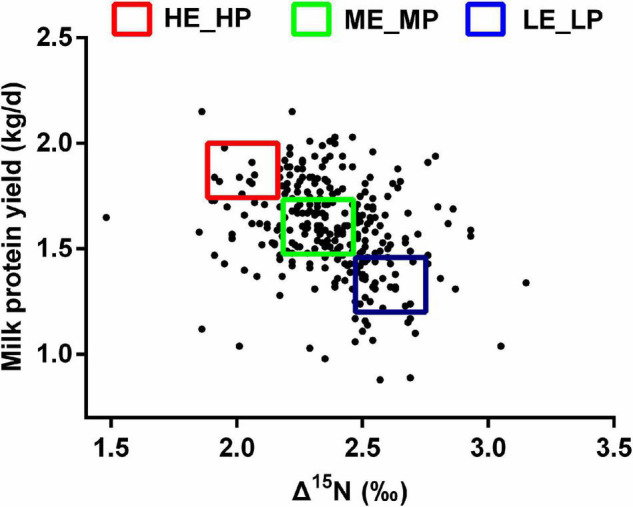
Scatter plot of Δ^15^N and milk protein yield among the HE_HP cows, ME_MP cows, and LE_LP cows.

The experiment procedures were approved by the animal care committee (approval number: ISA2020-82). A total of 284 healthy lactating Holstein dairy cows (parity = 2; days in milk = 48 ± 1, mean ± SEM) were selected from 4,086 dairy cows in a commercial dairy farm. The dairy cows were fed an identical diet (Supplementary Table 1) and had free access to water. Milk yield was recorded for a month, and milk samples were collected on the last day at a volume ratio of 4:3:3 homologized to the morning (0500h), afternoon (1400h), and evening (1700h). Milk protein was tested by infrared analysis using near-infrared spectroscopy. Milk protein yield was calculated using milk protein content multiplied by the milk yield. Milk urea nitrogen was measured with the Urea Assay Kit (Nanjing Jiancheng Bioengineering Institute, Nanjing). Blood was collected using a 10-ml heparin anticoagulant tube from the caudal vein within 2 h after morning feeding. The blood samples were centrifuged at 4,000 × *g* for 5 min to obtain plasma and stored at −20°C. The plasma ^15^N and diet ^15^N were determined by Elemental Analysis-Isotope Ratio Mass (Isoprime100, England). Δ^15^N was calculated using δ^15^N plasma - δ^15^N diet (Oenema and Tamminga, 2005). Based on Δ^15^N and MPY, 7 high NUE [HE_HP: 1.9‰ < Δ^15^N < 2.2‰; 1.7 kg/day < MPY < 2.0 kg/day, days in milk (DIM) = 59 ± 9] dairy cows, 7 medium NUE (ME_MP: 2.2‰ < Δ^15^N < 2.4‰; 1.4 kg/day < MPY < 1.7 kg/day, DIM = 54 ± 15) dairy cows, and 7 low NUE (LE_LP: 2.4 ‰ < Δ^15^N < 2.7 ‰; 1.0 kg/day < MPY < 1.4 kg/day, DIM = 63 ± 9) dairy cows were selected (Figure 1). The rumen liquid was collected within 2 h after the morning feeding using oral stomach and tube and stored in liquid nitrogen immediately.

### Rumen Fermentation Parameter Detection

The rumen liquid was centrifuged at 13,000 × *g* at 4°C for 5 min to obtain the supernatant. Then, 600 μl of the supernatant was mixed with 50 μl of 25% metaphosphoric acid and was centrifuged at 12,000 × *g* for 20 min to obtain the supernatant for the analysis of ammonia nitrogen and VFAs. The ammonia nitrogen concentrations were determined using phenol-sodium hypochlorite colorimetric analysis (Broderick and Clayton, 1997). The proportion of VFAs was determined using GC (Agilent, 7890A) (Liang et al., 2017).

### DNA Extraction and 16S rRNA Gene Sequencing

Total DNA of rumen liquid was extracted from the rumen liquid of 21 dairy cows using cetyl trimethyl ammonium bromide (Coolaber, Beijing) plus bead-beating method (Minas et al., 2011). The qualities and quantities of the DNA samples were measured using NanoDrop (Thermo, Beijing). The DNA was amplified using primers 341F (5′-CCTAYGGGRBGCASCAG-3′) and 806R (5′-GGACTACNNGGGTATCTAAT-3′), which target the V3–V4 region of the bacterial 16S rRNA gene. The reactions were performed in a 50-μl reaction mixture containing 2 μl DNA, 3.0 μl of each primer, 1.0 μl of dNTP, 0.25 of DNA polymerase (Takara, Dalian), 5 μl of 10 × PCR buffer, and 38.75 of ddH_2_O. The PCR cycling procedures were as follows: 95°C for 5 min, 30 cycles of 94°C for 30 s, 50°C for 30 s, 72°C for 30 s, and followed by 72°C for 15 min. The amplicon sequencing library was constructed using TruSeq Nano DNA LT Library Prep Kit (Illumina, America). After the library quality control was finished, the library was sequenced on an Illumina HiSeq platform (Illumina, America).

### Bioinformatic Analysis

Reads were removed primers, demultiplexed, and quality-filtered using Vsearch (Rognes et al., 2016). The reads were truncated at any site of >3 sequential bases receiving a quality score of <Q20, and the reads with <75% (of total read length) consecutive high-quality base calls were removed. Operational taxonomic units (OTU) were analyzed using QIIME 2.0 and clustered at 97% similarity using Vsearch (Haas et al., 2011). OTUs were filtered based on the number of samples in an out <10 and aligned using MAFFT. The phylogenetic tree was constructed using FASTTREE. OTUs were further conducted for species annotation using SKLEARN based on SILVA-138 database. Data rarefying was performed using the minimum size of reads in a sample. The statistical bioinformatics analysis of this study was performed using MicrobiomeAnalyst (Chong et al., 2020). The OTU-level alpha diversity of rumen bacteria was obtained using Chao1 index, ACE index, Shannon index, and Simpson index. The OTU-level beta diversity was performed based on weighted UniFrac and visualized by principal coordinate analysis (PCoA) using the phyloseq package in R (McMurdie and Holmes, 2013). Linear discriminant analysis effect size (LEfSe) was performed on non-parametric factorial Kruskal–Wallis sum-rank test to identify bacteria with significantly different abundances in the three groups (Segata et al., 2011), followed by linear discriminant analysis (LDA) to calculate the effect size of each differently abundant bacteria. The heat map of bacteria and MPY, Δ^15^N was generated using RevolverMaps.^1^

### Statistical Analysis

Lactation performance (DIM, milk yield, milk protein, milk urea nitrogen, and milk protein yield), Δ^15^N, rumen fermentation parameter concentrations (VFA, NH_3_–N), and alpha diversity indices of HE_HP cows, ME_MP cows, and LE_LP cows were analyzed using one-way ANOVA. The relative abundances of bacteria and alpha diversity indices among the three groups were compared using Kruskal–Wallis test. Dunn’s multiple-comparisons test was used for multiple comparisons of means among the three groups. Statistical analysis was performed using GraphPad Prism 7.0, with *P* < 0.05 considered as statistical significance. LEfSe was used to compare the relative abundances of rumen bacteria in HE_HP cows, ME_MP cows, and LE_LP cows, and significant differences were considered by a LDA score >2 and *P* < 0.05. Correlation analysis between the specific bacteria and NUE was performed using Spearman’s correlation.

## Results

### Production and Rumen Volatile Fatty Acid Related to Nitrogen Utilization Efficiency

As to the selected 21 dairy cows, the milk yield was 44.37 ± 1.75 kg/day (mean ± SEM), the MPY of 21 dairy cows was 1.50 ± 0.06 kg/day, Δ^15^N was 2.30 ± 0.05 ‰, the composition of acetate was 60.22 ± 0.60%, propionate was 27.15 ± 0.57%, isobutyrate was 0.47 ± 0.02%, butyrate was 9.79 ± 0.22%, isovalerate was 0.84 ± 0.04%, valerate was 1.52 ± 0.08%, and NH_3_–N was 8.85 ± 0.67 mg/dl. Milk yield and MPY in HE_HP were the highest among the three groups (*P* < 0.01) and in LE_LP cows were the lowest. In contrast, Δ^15^N in HE_HP cows was the lowest and in LE_LP cows was the highest (*P* < 0.01; Table 1). The DIM, milk protein, and milk urea nitrogen showed no significance among the HE_HP cows, ME_MP cows, and LE_LP cows (*P* > 0.05). Furthermore, the ruminal levels of NH_3_–N and the proportion of butyrate and valerate were not different among the three groups (*P* > 0.05). However, the HE_HP cows exhibited a significantly lower concentration of acetate (58.89%) and a higher concentration of propionate (28.87%) compared to LE_LP cows (*P* < 0.05).

**TABLE 1:** The production, nitrogen utilization efficiency indexes, and rumen fermentation parameters of dairy cows.

	Group			SEM	*P*-value
	
	HE_HP	ME_MP	LE_LP		
Milk yield (kg/day)	51.89a	46.06b	35.16c	1.75	< 0.01
Milk protein (%)	3.49	3.31	3.39	0.06	0.49
Milk urea nitrogen (mg/dl)	14.80	14.90	15.14	0.38	0.93
Milk protein yield (kg/day)	1.79a	1.52b	1.19c	0.06	< 0.01
Δ^15^N (‰)	2.05c	2.30b	2.54a	0.05	< 0.01
**Ruminal VFA composition (%)**					
Acetate	58.89b	59.11b	62.67a	0.60	< 0.01
Propionate	28.87a	28.25a	24.34b	0.57	< 0.01
Isobutyrate	0.44	0.51	0.47	0.02	0.27
Butyrate	9.34	9.87	10.15	0.22	0.21
Isovalerate	0.79	0.86	0.86	0.04	0.64
Valerate	1.66	1.40	1.49	0.08	0.57
A:P ratio	2.05b	2.10b	2.60a	0.22	< 0.01
Ruminal NH_3_–N (mg/dl)	7.11	10.25	9.17	0.67	0.15

*Lowercase letters indicate different shoulder markers of peer data. A:P ratio, ratio of acetate to propionate; VFA, volatile fatty acid.*

### Rumen Bacterial Diversity Related to Nitrogen Utilization Efficiency

**FIGURE 2:**
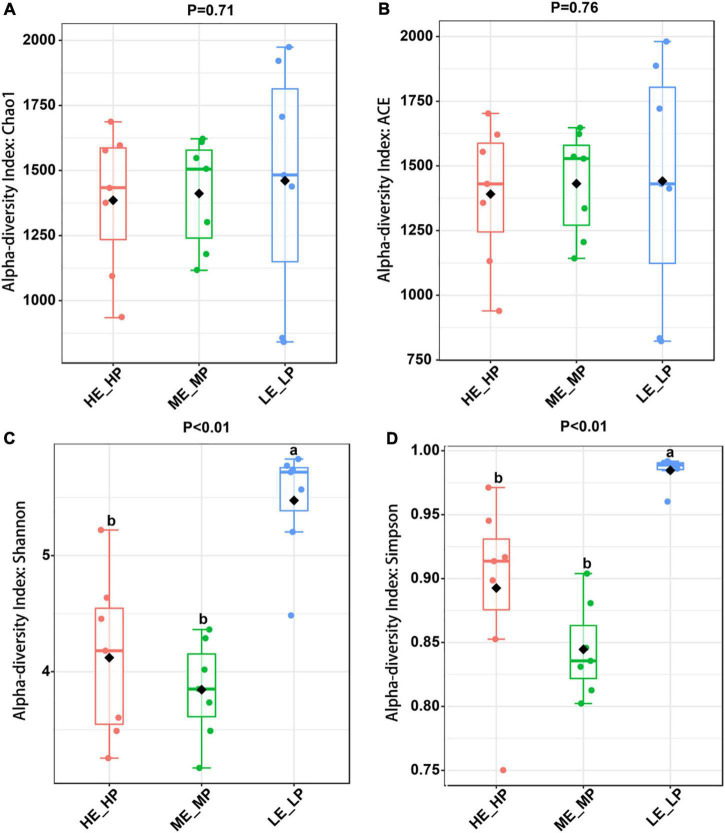
Alpha diversity analysis of rumen microbiota in different nitrogen utilization efficiency groups. **(A)** Chao 1 index among the HE_HP cows, ME_MP cows, and LE_LP cows. **(B)** ACE index among the HE_HP cows, ME_MP cows, and LE_LP cows. **(C)** Shannon index among the HE_HP cows, ME_MP cows, and LE_LP cows. **(D)** Simpson index among the HE_HP cows, ME_MP cows, and LE_LP cows.

**FIGURE 3:**
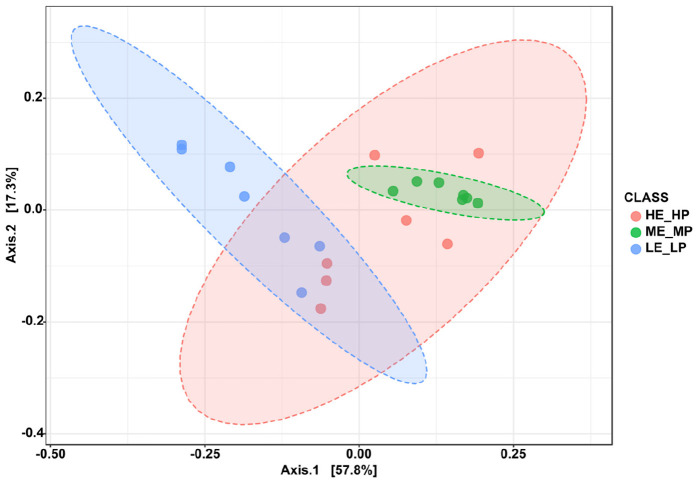
Principal coordinate analysis of bacteria beta diversity at species level among the HE_HP cows, ME_MP cows, and LE_LP cows.

Amplicon sequencing of the 16S rRNA generated a total of 1,647,027 high-quality sequences and 8,987 OTUs across 21 samples (Supplementary Figure 1). The rarefaction curves revealed that the sequencing depth was sufficient to cover most species of rumen bacteria (Supplementary Figure 2). The Chao1 index and ACE index (*P* > 0.05) were not significantly different in HE_HP cows, ME_MP cows, and LE_LP cows (Figures 2A,B). The Shannon index and Simpson index (*P* < 0.01) were significantly higher in LE_LP cows compared to HE_HP cows and ME_MP cows (Figures 2C,D). The PCoA based on weighted UniFrac distances showed that the bacterial composition between the HE_HP and LE_LP groups was different (PERMANOVA, *P* < 0.01), while the ME_MP group had no significant difference compared to the HE_HP group (Figure 3), and the ME_MP group had the most homogeneous community distribution compared to the other two groups.

### Different Rumen Bacterial Taxa in Dairy Cows With Different Nitrogen Utilization Efficiency

**FIGURE 4:**
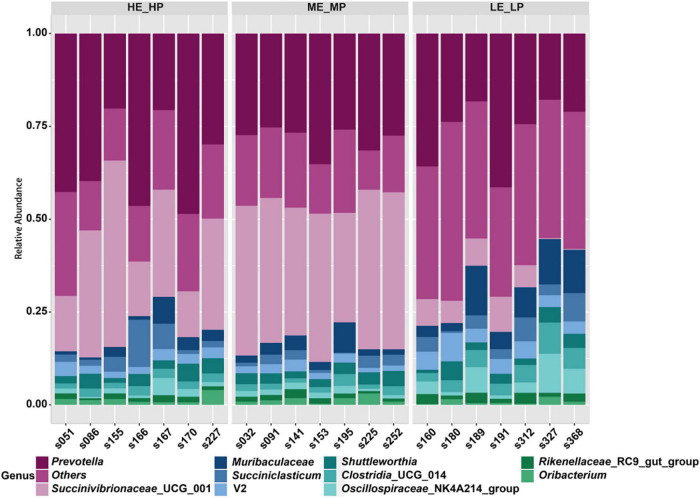
Relative abundances of rumen microbiota at the genus level among the HE_HP cows, ME_MP cows, and LE_LP cows.

A total of 24 bacterial phyla were identified; the predominant bacterial phyla were Bacteroidota (41.24 ± 8.02%), Firmicutes (30.25 ± 11.96%), and then Proteobacteria (25.78 ± 14.97%) (Supplementary Figure 3). At the family level, the predominant bacterial families were Prevotellaceae (32.76 ± 9.4%), Succinivibrionaceae (23.48 ± 16.25%), and Lachnospiraceae (12.84 ± 4.79%) (Supplementary Figure 4). At the genus level, a total of 326 genera were identified; *Prevotella* (29.85 ± 9.53%), *Succinivibrionaceae*_UCG_001 (23.42 ± 16.38%), *Muribaculaceae* (4.61 ± 3.95%), and *Succiniclasticum* (3.35 ± 2.91%) were the most abundant genera (Figure 4).

**FIGURE 5:**
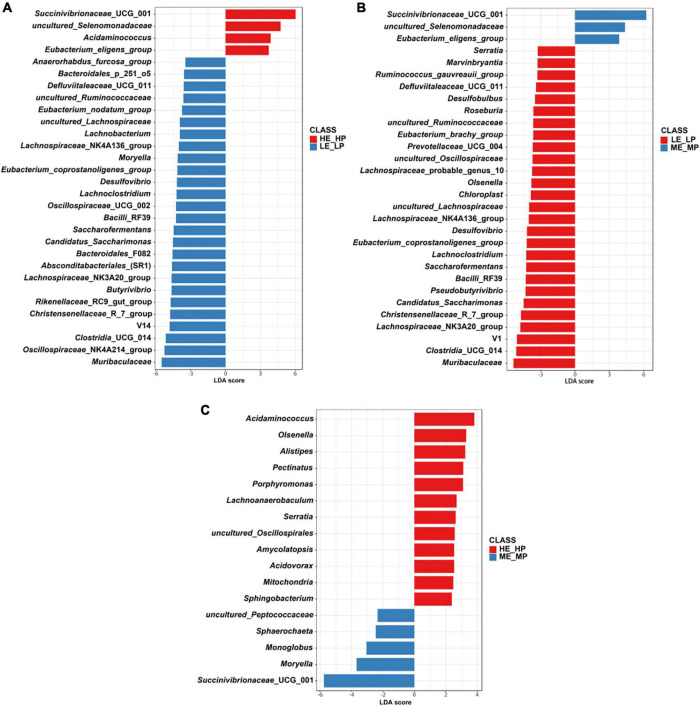
Comparison of the relative abundances of rumen bacteria among the HE_HP cows, ME_MP cows, and LE_LP cows by linear discriminant analysis effect size (*P* < 0.05; linear discriminant analysis, >2). **(A)** Different rumen bacteria between HE_HP cows and LE_LP cows. **(B)** Different rumen bacteria between LE_LP cows and ME_MP cows. **(C)** Different rumen bacteria between HE_HP cows and ME_MP cows.

The different abundant bacteria at the genus level among the HE_HP cows, ME_MP cows, and LE_LP cows were revealed by LEfSe (Figure 5). The relative abundance of *Succinivibrionaceae*_UCG_001, uncultured_Selenomonadaceae, and *Acidaminococcus* was higher in HE_HP cows (*P* < 0.05). Moreover, *Clostridia*_UCG_014, *Saccharofermentans*, *Bacilli*_ RF39, and *Desulfovibrio* were higher in LE_LP cows (*P* < 0.01). There were differences in the abundances of bacterial taxa among the three groups (Table 2). Meanwhile, LEfSe was used to screen the different OTUs (Supplementary Table 2). The genera corresponding to OTUs with significant differences included *Succinivibrionaceae*_UCG_001, *Shuttleworthia*, *Clostridia*_UCG_014, uncultured_*Selenomonadaceae*, *Lachnos piraceae*_NK3A20_group, *Christensenellaceae*_R-7_group, *Desu lfovibrio, Saccharofermentans*, *Allisonella*, and *Eubact erium_eligens*_group (*P* < 0.05), and there was 20% less genus Succinivibrionaceae_UCG_001 in LE_LP cows than in the other two groups. Moreover, the most differently abundant OTUs belonged to the phylum of Firmicutes.

**TABLE 2:** Relative abundances of specific bacteria in dairy cows with different nitrogen utilization efficiency.

Genus	Relative abundance (%)			SEM	*P*-value
	
	HE_HP	ME_MP	LE_LP		
Succinivibrionaceae_UCG_001	26.43a	38.33a	5.19b	3.54	< 0.01
uncultured_Selenomonadaceae	1.42a	1.10a	0.60b	0.17	0.04
*Acidaminococcus*	0.16a	0.03b	0.03b	0.03	0.03
*Eubacterium_eligens*_group	0.14*ab*	0.21a	0.08b	0.02	0.02
*Clostridia*_UCG_014	2.09b	1.85b	4.57a	0.38	< 0.01
Lachnospiraceae_NK3A20_group	0.86*ab*	0.40b	1.60*a*	0.22	< 0.01
*Candidatus_Saccharimonas*	0.34*ab*	0.30b	0.93a	0.09	< 0.01
*Saccharofermentans*	0.32b	0.39b	0.81a	0.07	0.03
*Bacilli*_RF39	0.29b	0.26b	0.66a	0.05	< 0.01
*Desulfovibrio*	0.19b	0.17b	0.48a	0.05	< 0.01
*Lachnoclostridium*	0.20*ab*	0.16b	0.51a	0.06	< 0.01
uncultured_Lachnospiraceae	0.13*ab*	0.08b	0.27a	0.03	0.03
Lachnospiraceae_NK4A136_group	0.18b	0.22*ab*	0.42a	0.04	0.03
*Eubacterium_coprostanoligene*s_group	0.20b	0.17b	0.47a	0.04	< 0.01
Defluviitaleaceae_UCG_011	0.06b	0.05b	0.14a	0.01	< 0.01
*Olsenella*	0.04*ab*	0.02b	0.14a	0.02	< 0.01
Uncultured_Ruminococcaceae	0.03b	0.03b	0.14a	0.01	< 0.01
Lachnospiraceae_probable_genus_10	0.05b	0.06*ab*	0.18a	0.02	0.02

*The bacteria with relative abundances were greater than 0.01% among HE_HP cows, ME_MP cows, and LE_LP cows. The different lowercase letters mean the significance (P < 0.05).*

### Relationships of Different Rumen Bacteria With Δ^15^N and Milk-Protein-Yield

**FIGURE 6:**
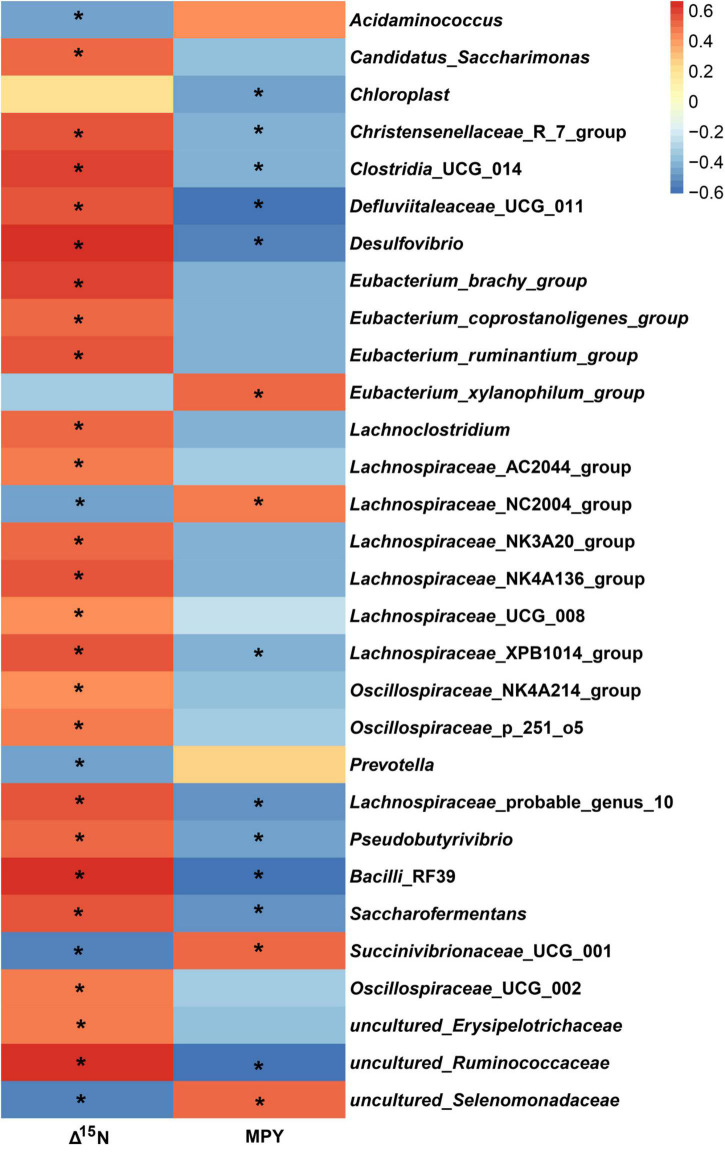
Spearman correlations between specific bacterial genera, Δ^15^N, and MPY. The asterisk means significant difference (*P* < 0.05).

**FIGURE 7:**
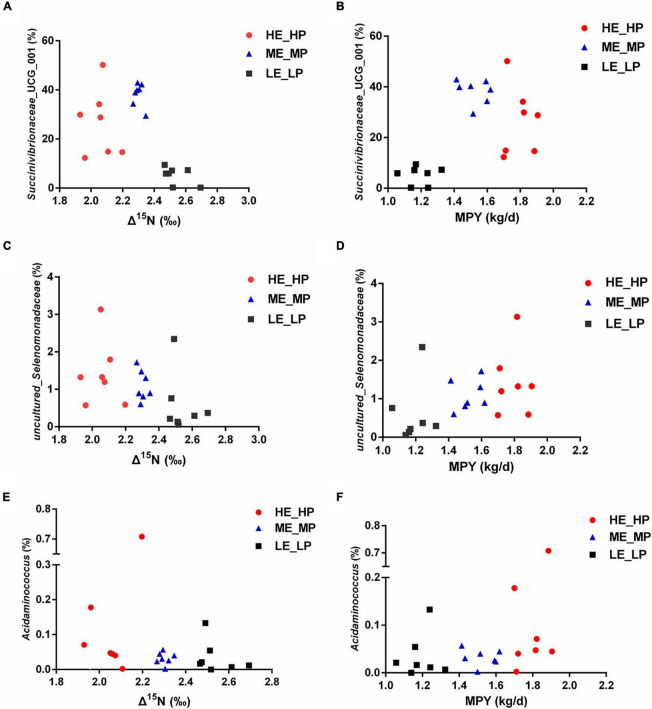
Relative abundances of rumen bacteria and Δ^15^N and milk protein yield (MPY). **(A)** Scatter plot of Succinivibrionaceae_UCG_001 and Δ^15^N. **(B)** Scatter plot of Succinivibrionaceae_UCG_001 and MPY. **(C)** Scatter plot of uncultured_Selenomonadaceae and Δ^15^N. **(D)** Scatter plot of uncultured_Selenomonadaceae and MPY. **(E)** Scatter plot of *Acidaminococcus* and Δ15N. **(F)** Scatter plot of *Acidaminococcus* and MPY.

In the study, in all of the 326 encountered genera, the relative abundance of 127 bacterial taxa was >0.01%. We analyzed the 127 bacterial taxa and Δ^15^N and MPY using Spearman’s correlation. The correlation analysis revealed that 32 bacterial taxa were significantly correlated with Δ^15^N and MPY (Figure 6). The results showed that Δ^15^N and MPY possessed remarkable relationships with bacterial taxa, including five positive relationships and 30 negative relationships (*P* < 0.05). Among them, *Succinivibrionaceae*_UCG_001, uncultured_Selenomonadaceae, *Acidaminococcus*, *Eubacterium_xylanophilum*_group, and *Lachnospiraceae*_NC2004_group were positively correlated with MPY and negatively correlated with Δ^15^N. The scatter plots of the specific bacteria and Δ^15^N and MPY are shown in Figure 7, which shows that the same genus of dairy cows with different NUE had different abundance levels, but *Clostridia*_UCG_014, *Saccharofermentans*, *Bacilli*_RF39, and *Desulfovibrio* were negatively correlated with MPY and positively correlated with Δ^15^N (*P* < 0.05).

## Discussion

At present, the most effective way to improve the NUE of dairy cows is to match the dietary nitrogen content and energy supply during rumen fermentation by changing the diet composition (Reid et al., 2015). In addition, maximum NUE is usually achieved at the cost of some loss-of-production performance, but it can be achieved by understanding the key mechanisms that regulate nitrogen metabolism. The indexes commonly used to evaluate the NUE of dairy cows include blood urea nitrogen, milk urea nitrogen, milk protein yield, and Δ^15^N (Kohn et al., 2005; Burgos et al., 2007; Warinner and Tuross, 2010). The Δ^15^N, compared to other indicators, can overcome the differences at an individual level and is better for large groups of cows (Cantalapiedra-Hijar et al., 2016). In recent years, several studies have proved Δ^15^N as a biomarker to assess NUE, and many scholars have pointed out that Δ^15^N was negatively correlated with NUE and rumen microbial protein synthesis efficiency (Cheng et al., 2011, 2014; Cantalapiedra-Hijar et al., 2015). MPY is an important index to measure the lactating performance of dairy cows, which is a new biomarker for high milk protein and high yield (Wu et al., 2018; Xue et al., 2019). However, the MPY is not necessarily high when NUE is high, which is not in line with our aims of efficiency and production. Thus, the cows were divided into HE_HP cows, ME_MP cows, and LE_LP cows according to Δ^15^N and MPY, and the rumen fermentation indexes were compared. The proportion of propionate and the ratio of acetate to propionate, which is mainly involved in carbohydrate metabolism, were higher in HE_HP cows and ME_MP cows than in LE_LP cows, suggesting that propionate had a closed correlation with the rumen nitrogen metabolism. The propionate produced in the rumen was absorbed and transferred to glucose which provided energy for milk protein synthesis. Similarly, Xue et al. (2019) also found that high-milk-production and high-protein-content cows had significantly more ruminal propionate compared to those with low milk production and protein content. However, ruminal NH_3_–N showed no significant changes among the three groups, which may be due to its low variation and levels (<11 mg/dl).

The rumen bacteria play an important role in dietary nutrient digestion and absorption. Furthermore, we detected that the diversity of rumen bacteria and specific rumen bacterial taxa contributed to NUE among the HE_HP cows, ME_MP cows, and LE_LP cows. The alpha diversity indices of Shannon and Simpson showed a lower bacterial diversity in HE_HP cows. Xue et al. (2020) compared the rumen microbial structure of high-MPY cows and low-MPY cows and found that the ruminal microbial richness in high-MPY cows was lower. It has been reported that the higher feed efficiency of dairy cows possessed a lower diversity, indicating that HE_HP cows can have higher feed efficiency than ME_MP cows and LE_LP cows and also suggesting that HE_HP cows have higher ruminal fermentation efficiency (Li and Guan, 2017). A lower diversity of the rumen microbiota has been identified in cows with a higher milk yield (Shabat et al., 2016), which is consistent with our results.

We revealed the difference in the relative abundances of rumen bacteria that may be associated with NUE. The family Succinivibrionaceae was higher in HE_HP cows and ME_MP cows than in LE_LP cows. Succinivibrionaceae, including 6 genera (*Anaerobiospirillum*, Succinivibrionaceae_UCG_001, *Ruminobacter*, Succinivibrionaceae_UCG_002, *Succinimonas*, and *Succinivibrio*), was the most active rumen bacterial family in beef (Li and Guan, 2017). Succinivibrionaceae is one of the contributors to producing succinate, and succinate can be used by other microorganisms to synthesize propionate and valerate, and the abundance of Succinivibrionaceae has been reported to be related to energy metabolism and methane emission (Wallace et al., 2015). Xue et al. (2020) pointed out that the methanogens in rumen had a lower relative abundance in high-MPY dairy cows, suggesting that these cows can produce less methane and sufficiently satisfy the higher performance of milk protein and milk yield. Succinivibrionaceae_UCG_001 composed the core rumen microbiota in young cattle and is the most abundant genus of the phylum Proteobacteria (Wang et al., 2019). In the study, we found that *Succinivibrionaceae*_UCG_001 was higher in HE_HP cows and ME_MP cows than in LE_LP cows, indicating that HE_HP cows and ME_MP cows have higher levels of propionate, a major substrate for gluconeogenesis, and it is enough to supply glucose for milk production to influence NUE. Furthermore, there are 6 OTUs belonging to *Succinivibrionaceae*_UCG_001 in the top 50 specific OTUs. It has been reported that *Succinivibrionaceae*_UCG_001 was negatively correlated with the ratio of acetate to propionate (Cao et al., 2020). The Spearman correlation analysis showed that *Succinivibrionaceae*_UCG_001 was positively related with MPY and negatively with Δ^15^N, which supported the important contribution of *Succinivibrionaceae* to nitrogen metabolism in dairy cows. Our results revealed that the production of propionate and acetate might affect the glucose supply, which could influence the NUE of dairy cows. The uncultured_*Selenomonadaceae* also had more abundance in HE_HP cows and ME_MP cows, and it was positively correlated with MPY and negatively correlated with Δ^15^N. It indicated that the following research on the uncultured_*Selenomonadaceae* was needed to reveal its contribution to NUE. Moreover, *Acidaminococcus* was higher in HE_HP cows than in ME_MP cows and LE_LP cows, and it was negatively related with Δ^15^N. *Acidaminococcus* has been demonstrated to utilize amino acids as the sole energy source to produce acetic and butyric acids, which were negatively associated with metabolic diseases such as obesity (Rogosa, 1969; Li et al., 2021). The effect of *Acidaminococcus* on ruminant NUE was still unclear, and more culturing studies are needed to find out its role in nitrogen metabolism.

However, the results also revealed that the relative abundances of *Clostridia*_UCG_014, *Saccharofermentans*, *Bacilli*_RF39, and *Desulfovibrio* were higher in LE_LP cows than in HE_HP cows and ME_MP cows (relative abundance, >0.1%). Thus, it might demonstrate that these four bacteria were negatively associated with NUE. *Clostridia*_UCG_014 is a kind of pro-inflammatory bacteria (Wang et al., 2021), and its high abundance potentially led to the production of more proinflammatory metabolites to interfere with nitrogen metabolism. *Saccharofermentans* isolated from sludge treating brewery wastewater can ferment cellulose and starch to produce acetate (Chen et al., 2010; Dai et al., 2016). A recent study has indicated the higher abundance of *Saccharofermentans* in the rumen of cows with laminitis (Opdahl et al., 2018). Bacilli_RF39 was a novel Tenericutes lineage and reported in goats and the human gut (Zhang et al., 2015; Nayfach et al., 2019). Due to the loss of ability to biosynthesize fatty acids, it is difficult to degrade the cellular membranes of plants for dairy cows. *Desulfovibrio* can reduce sulfate to sulfide compounds, which can block the oxidation of short-chain fatty acids in the rumen and compete with Firmicutes for energy substance and carbon source (Jia et al., 2012). In our study, *Saccharofermentans*, *Bacilli*_RF39, and *Desulfovibrio* were negatively correlated with MPY and were positively correlated with Δ^15^N, suggesting that those bacteria had a negative function in improving the rumen nitrogen metabolism. Although Δ^15^N is a good biomarker for NUE, it is not the actual NUE. Here we just found out the presence of related bacterial species to Δ^15^N. The activity and function of these bacteria contributing to the NUE which are more direct and important need to be revealed in the future. More productive data, e.g., individual feed intake, are beneficial to reveal the closely related rumen microbes to NUE, which needs to be addressed in future studies. In addition, microbes such as archaea, protozoa, and anaerobic fungi also had roles in ruminal nitrogen metabolism, but we were solely focused on the bacteria community in this study. Therefore, the contribution of archaea, protozoa, and anaerobic fungi on nitrogen utilization efficiency has to be revealed in the following studies.

## Conclusion

The relative abundances and diversity of rumen bacteria in dairy cows were clearly different among high- and low-NUE cows. The relative abundances of *Succinivibrionaceae*, *Selenomonadaceae*, and *Acidaminococcus* were higher in the rumen of high-NUE cows, which positively related with MPY and negatively with Δ^15^N. The study indicated that ruminal bacteria was a potential regulation target to enhance the nitrogen utilization of dairy cows. However, the activity and function of these bacteria contributing to the NUE need to be studied in the future.

## Data Availability Statement

The original contributions presented in the study are included in the article/Supplementary Material, further inquiries can be directed to the corresponding authors.

## Ethics Statement

The animal study was reviewed and approved by Animal Care and Use Committee for Livestock of the Institute of Animal Sciences, Chinese Academy of Agricultural Sciences (approval number: ISA2020-82).

## Author Contributions

MnL contributed to conceptualization, software, data curation, and writing—original draft. HZ performed data curation. MgL took charge of the software. NZ was in charge of supervision. SZ contributed to writing—review and editing. JW was in charge of administration. All authors contributed to the article and approved the submitted version.

## Conflict of Interest

The authors declare that the research was conducted in the absence of any commercial or financial relationships that could be construed as a potential conflict of interest.

## Publisher’s Note

All claims expressed in this article are solely those of the authors and do not necessarily represent those of their affiliated organizations, or those of the publisher, the editors and the reviewers. Any product that may be evaluated in this article, or claim that may be made by its manufacturer, is not guaranteed or endorsed by the publisher.

## Footnotes

1
https://www.revolvermaps.com
