# Supplementary material for: Contribution of Ruminal Bacteriome to the Individual Variation of Nitrogen Utilization Efficiency of Dairy Cows
Source: Front Microbiol. 2022 Mar 18;13:815225. doi: 10.3389/fmicb.2022.815225 (PMC8975277; doi:10.3389/fmicb.2022.815225)
Supplement: Supplementary file 1 [file Data_Sheet_1.docx]

**Supplemental Files**

Table S1. Composition and nutrient contents of the diet (% of DM)

| Item | Value |
| --- | --- |
| Ingredients |  |
| Corn silage | 49.22 |
| Alfalfa hay | 5.41 |
| Beet meal | 5.42 |
| DDGS^1^ | 1.48 |
| Corn slice | 13.04 |
| Corn meal | 6.65 |
| Soybean meal | 10.09 |
| Cottonseed | 3.69 |
| Molasses | 1.97 |
| Fat powder | 0.74 |
| Premix^2^ | 2.29 |
| Chemical composition (% of DM) |  |
| Crude protein | 17.00 |
| Starch | 27.00 |
| NDF^3^ | 27.00 |
| ADF^4^ | 16.80 |

^1^DDGS, distillers’ dried grains with solubles; ^2^The premix composition as following：Vitamin A 120000-360000 IU, Vitamin D330000-80000 IU, Vitamin E 800 mg, Mn 1000-3000 mg, Zn 1500-4000 mg, Cu 100-300 mg, Co 15-45 mg, I 15-45 mg, Se 6-18 mg, Mg 30000-80000 mg; ^3^NDF, neutral detergent fiber; ^4^ADF, acid detergent fiber.

Table S2. The top 15 significantly different OTUs among HE_HP cows, ME_MP cows and LE_LP cows.

| OTU | Relative abundance (%) | | | SEM P value | |
| --- | --- | --- | --- | --- | --- |
|  | HE_HP ME_MP LE_LP | | |  |  |
| Succinivibrionaceae_UCG_001(OTU-1) | 26.08^a^ | 37.81^a^ | 5.11^b^ | 3.61 | <0.01 |
| *Shuttleworthia*(OTU-2) | 0.84^a^ | 0.76^a^ | 0.20^b^ | 0.12 | <0.01 |
| *Eubacterium_ruminantium*_group(OTU-3) | 0.38 | 0.48 | 0.78 | 0.07 | 0.06 |
| *Clostridia*_UCG_014(OTU-4) | 0.31^b^ | 0.34^b^ | 0.69^a^ | 0.06 | <0.01 |
| *Clostridia*_UCG_014(OTU-5) | 0.45 | 0.38 | 0.25 | 0.06 | 0.03 |
| uncultured Selenomonadaceae(OTU-6) | 0.44^a^ | 0.43^a^ | 0.10^b^ | 0.06 | <0.01 |
| Lachnospiraceae_NK3A20_group(OTU-7) | 0.23^ab^ | 0.09^b^ | 0.40 ^a^ | 0.08 | <0.01 |
| Christensenellaceae_R-7_group(OTU-8) | 0.09^b^ | 0.14^ab^ | 0.34^a^ | 0.04 | 0.04 |
| *Shuttleworthia*(OTU-9) | 0.23 | 0.18 | 0.04 | 0.04 | 0.01 |
| *Desulfovibrio*(OTU-10) | 0.16b | 0.12b | 0.45a | 0.04 | <0.01 |
| *Saccharofermentans*(11) | 0.09^b^ | 0.14^ab^ | 0.27^a^ | 0.02 | 0.03 |
| *Prevotella*(OTU-12) | 0.15 | 0.19 | 0.26 | 0.03 | 0.05 |
| *Allisonella*(OTU-13) | 0.19a | 0.13^ab^ | 0.05^b^ | 0.02 | 0.02 |
| *Eubacterium_eligens*_group(OTU-14) | 0.19 | 0.16 | 0.09 | 0.02 | 0.01 |
| *Lachnospiraceae_probable*_genus_10(OTU-15) | 0.05 | 0.08 | 0.14 | 0.02 | 0.06 |


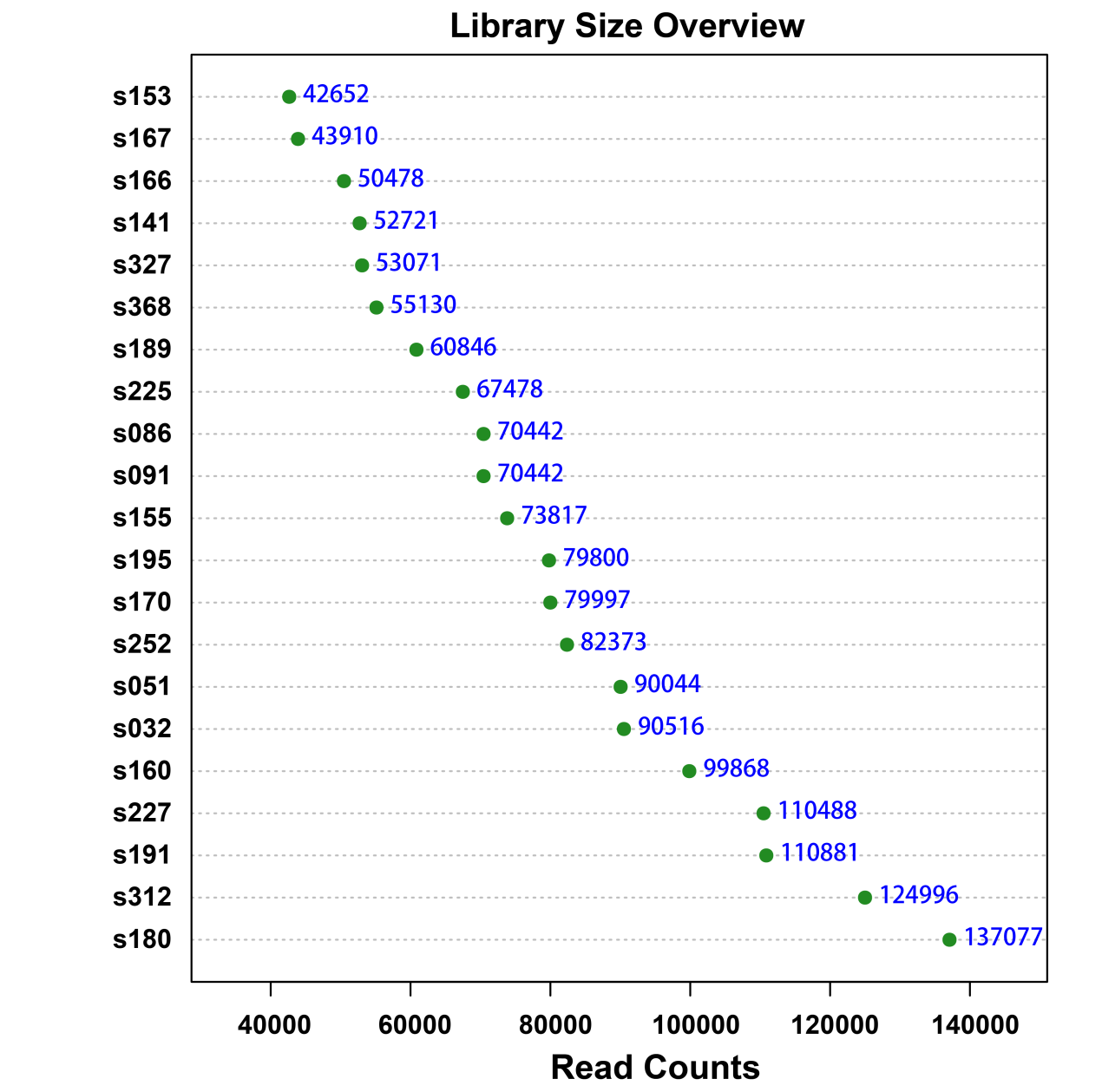


Figure S1. Reads counts of each sample among HE_HP cows, ME_MP cows, LE_LP cows.


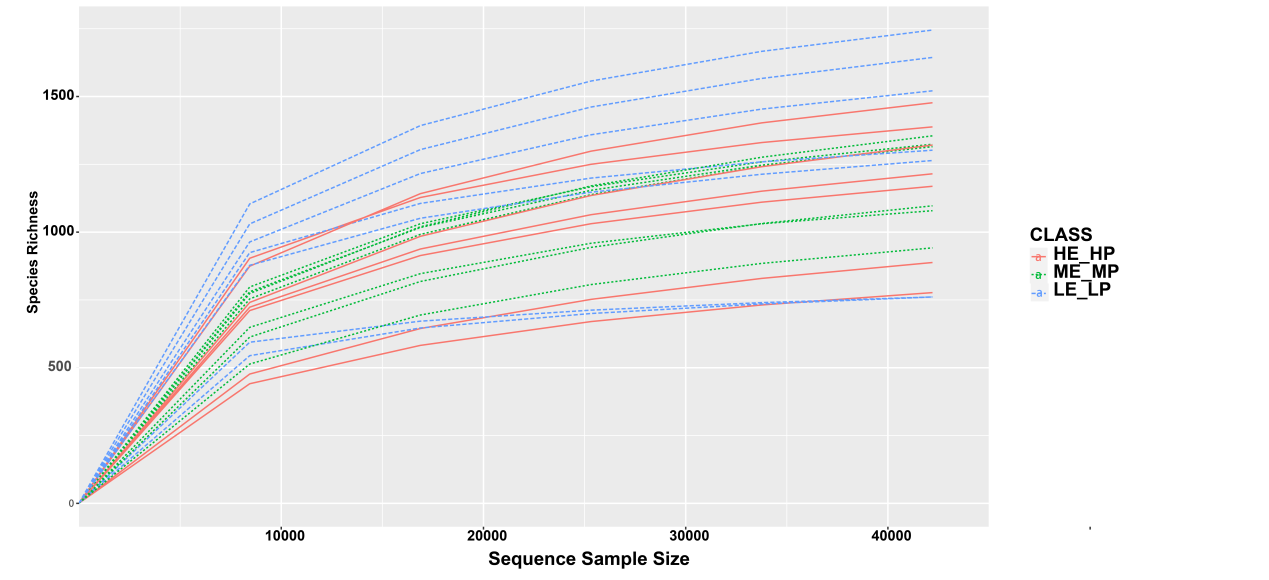


Figure S2. Rarefaction curve of each sample among HE_HP cows, ME_MP cows, LE_LP cows.


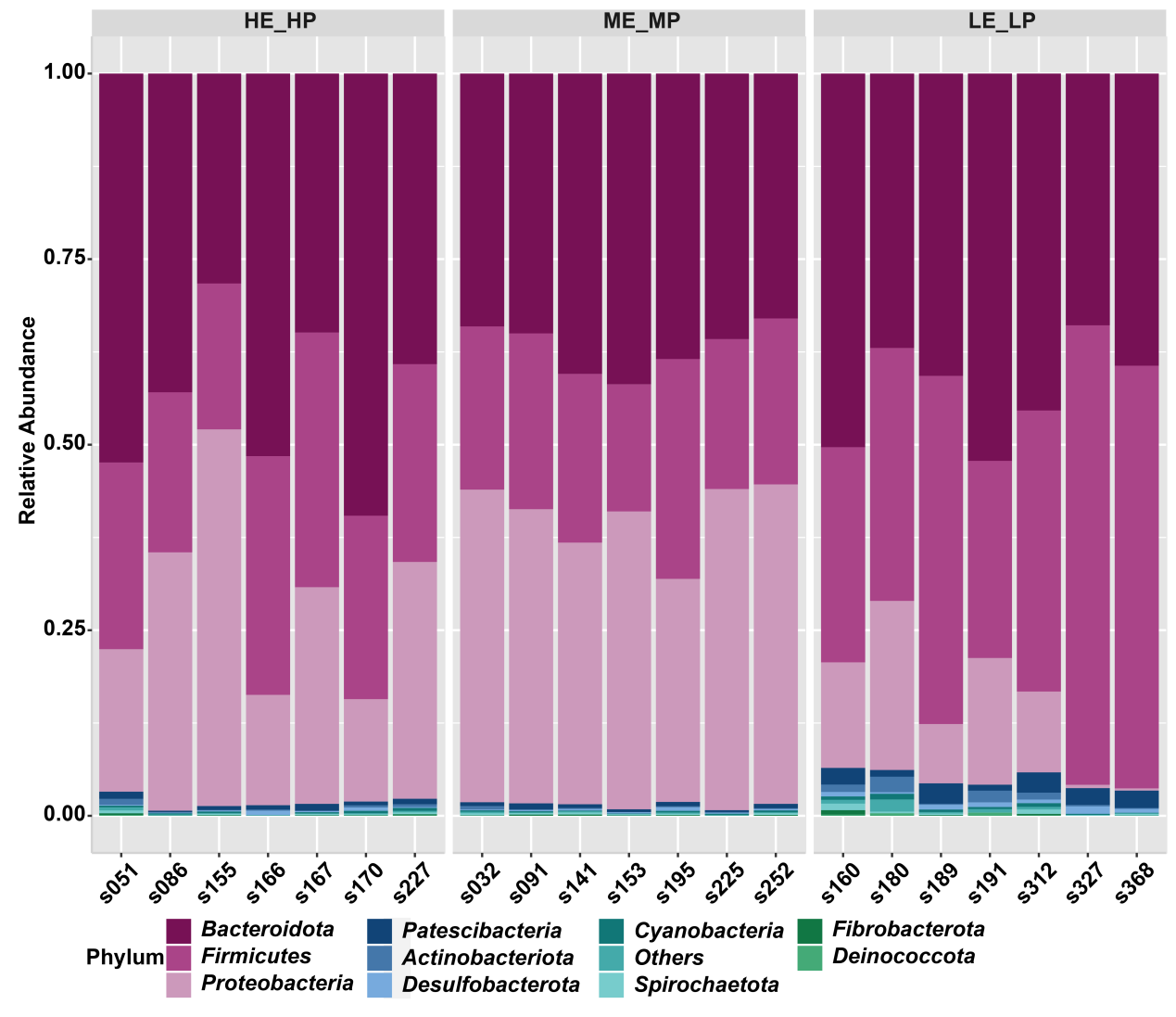


Figure S3. The relative abundances of rumen bacteria at phylum level among HE_HP cows, ME_MP cows and LE_LP cows.


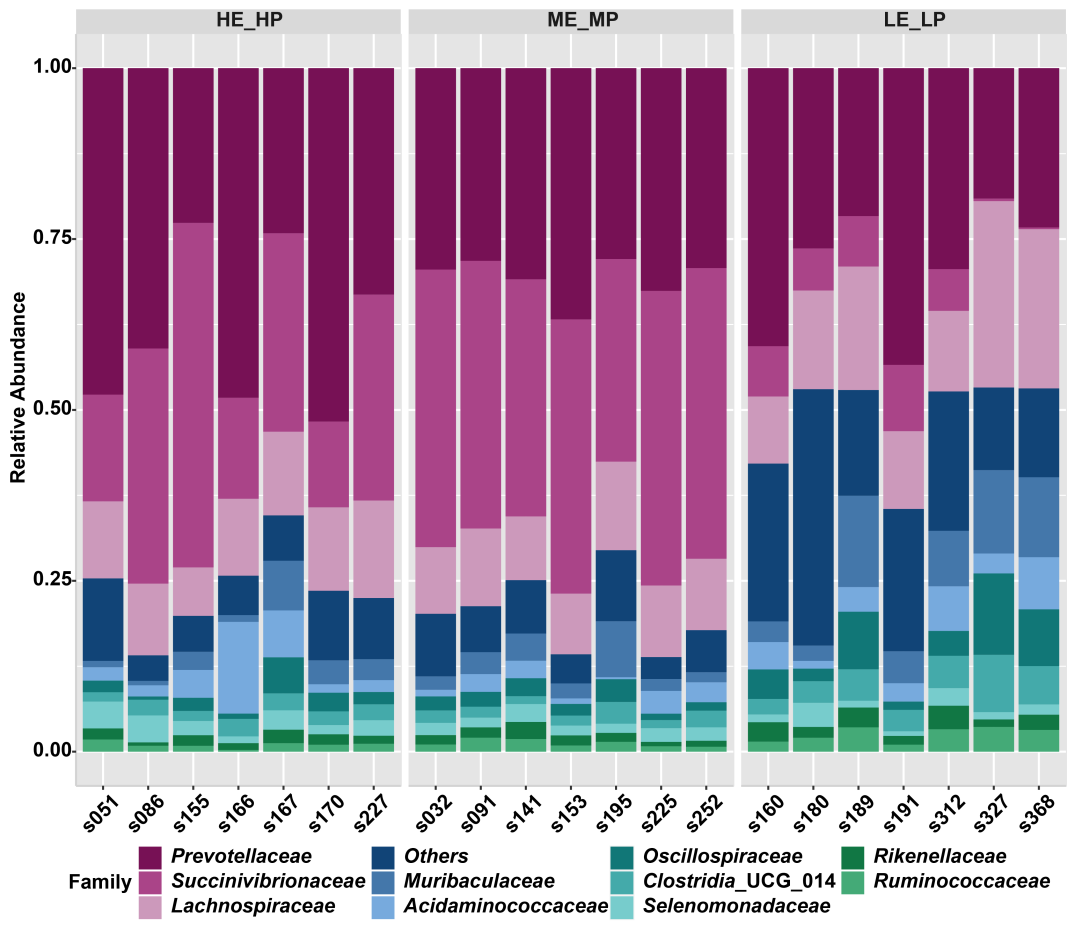


Figure S4. The relative abundances of rumen bacteria at family level among HE_HP cows, ME_MP cows and LE_LP cows.
